# Defining the role of TRPM4 in broadly responsive taste receptor cells

**DOI:** 10.3389/fncel.2023.1148995

**Published:** 2023-03-22

**Authors:** Debarghya Dutta Banik, Kathryn F. Medler

**Affiliations:** Department of Biological Sciences, University at Buffalo, Buffalo, NY, United States

**Keywords:** gustation, taste receptor cells (TRC), signal transduction, transient receptor potential melastatin 4 (TRPM4), broadly responsive taste cells

## Abstract

Peripheral taste receptor cells use multiple signaling pathways to transduce taste stimuli into output signals that are sent to the brain. We have previously identified a subpopulation of Type III taste cells that are broadly responsive (BR) and respond to multiple taste stimuli including bitter, sweet, umami, and sour. These BR cells use a PLCβ3/IP_3_R1 signaling pathway to detect bitter, sweet, and umami stimuli and use a separate pathway to detect sour. Currently, the downstream targets of the PLCβ3 signaling pathway are unknown. Here we identify TRPM4, a monovalent selective TRP channel, as an important downstream component in this signaling pathway. Using live cell imaging on isolated taste receptor cells from mice, we show that inhibition of TRPM4 abolished the taste-evoked sodium responses and significantly reduced the taste-evoked calcium responses in BR cells. Since BR cells are a subpopulation of Type III taste cells, they have conventional chemical synapses that require the activation of voltage-gated calcium channels (VGCCs) to cause neurotransmitter release. We found that TRPM4-dependent membrane depolarization selectively activates L-type VGCCs in these cells. The calcium influx through L-type VGCCs also generates a calcium-induced calcium release (CICR) via ryanodine receptors that enhances TRPM4 activity. Together these signaling events amplify the initial taste response to generate an appropriate output signal.

## Introduction

Chemicals in potential food items are detected by taste receptor cells that are located in taste buds within the oral cavity. Taste buds are comprised of distinct taste cell populations that vary in their functions, including the signaling pathways they express (Finger, 2005; Vandenbeuch and Kinnamon, 2009; Dutta Banik and Medler, 2021). Type I cells are thought to primarily function as support cells and have characteristics of glial cells (Lawton et al., 2000; Roper, 2007), while Type II cells detect bitter, sweet, and umami taste stimuli through the activation of a GPCR pathway (Miyoshi et al., 2001; Zhang et al., 2003). In Type II cells, this GPCR signaling activates phospholipase Cβ2 (PLCβ2) which causes calcium release from the internal stores via the inositol trisphosphate receptor 3 (IP_3_R3) to activate the monovalent cation selective transient receptor potential subfamily M members 4 and 5 (TRPM4/TRPM5) channels (Zhang et al., 2003; Dutta Banik et al., 2018). While Type II cells lack both voltage gated calcium channels (VGCCs) and conventional synapses (Yee et al., 2001; Clapp et al., 2004, 2006; Simon et al., 2006; Roper, 2007), activation of these TRP channels depolarizes the cell to generate action potentials that activate the calcium homeostasis modulator (Calhm1/Calhm3) channel which releases ATP onto the gustatory nerve (Gao et al., 2009; Taruno et al., 2013; Ma et al., 2018). Type III cells detect sour and salty stimuli through ionotropic receptors that depolarize the cell to generate action potentials which activate VGCCs and cause neurotransmitter release through conventional synapses (Yoshida et al., 2006; Huang et al., 2008; Gao et al., 2009; Chang et al., 2010; Lewandowski et al., 2016; Tu et al., 2018; Taruno et al., 2021).

We previously reported that a subset of Type III cells detects bitter, sweet, and/or umami stimuli using a PLCβ3/IP_3_R1 signaling pathway (Hacker et al., 2008; Dutta Banik et al., 2020). These cells are broadly responsive (BR) and can respond to taste stimuli from multiple taste modalities. Loss of PLCβ3 signaling in these cells causes severe taste deficits, indicating that they are important in taste transduction (Dutta Banik et al., 2020). How BR cells contribute to the transfer of taste information is still an open question. It may be that signals from Type II and BR taste cells are involved in coincidence detection such that both populations of taste cells must be activated to generate an output signal. Another possibility is that taste signals from Type II and BR cells are summed together to activate gustatory neurons. The BR cells may act as general detectors to alert the brain that a stimulus is present, while at higher concentrations, the Type II TRCs provide specific stimulus information. Further studies are needed to understand how this newly identified cell population contributes to taste.

The goal of this study is to better understand the signaling events that occur in BR taste cells. To date, the downstream components of the PLCβ3/IP_3_R1 signaling pathway in these BR cells have not been identified. One study using single taste cell transcriptome analysis reported that PLCβ3 and TRPM4 are co-expressed in a subset of Type III cells (Sukumaran et al., 2017) raising the possibility that they may be in the same signaling pathway. Since TRPM4 is a downstream component of the PLCβ2 signaling pathway in Type II cells (Dutta Banik et al., 2018), we hypothesized that it functions downstream of the PLCβ3 signaling pathway in BR cells. Using live cell imaging, we found that TRPM4 is a critical downstream target of PLCβ3 signaling in BR cells and links PLCβ3/IP_3_R1 signaling to VGCC activation.

## Materials and methods

### Mice

Animals were cared for in compliance with the University at Buffalo Institutional Animal Care and Use Committee. Most experiments were performed on C57Bl/6 mice with some experiments using the TRPM4-KO mice (Vennekens et al., 2007; Dutta Banik et al., 2018). All experiments were approved by the University at Buffalo Institutional Animal Care and Use Committee and were conducted following institutional and federal guidelines. At least three mice were used for each experiment. Mice were between 2 and 6 months of age, and both sexes were used for experiments.

### Taste receptor cell isolation

Taste receptor cells were harvested from circumvallate (CV) papillae of adult mice as previously described (Hacker and Medler, 2008; Hacker et al., 2008; Laskowski and Medler, 2009; Rebello and Medler, 2010; Starostik et al., 2010; Szebenyi et al., 2010; Dutta Banik et al., 2020). Briefly, mice were sacrificed using carbon dioxide and cervical dislocation. Tongues were removed, and an enzyme solution containing 0.7mg/mL Collagenase B (Roche, Basel, Switzerland), 3mg/mL Dispase II (Roche), and 1mg/mL Trypsin Inhibitor (Sigma-Aldrich, St. Louis, MO) was injected beneath the lingual epithelium. The tongues were incubated in oxygenated Tyrode’s solution for approximately 16 min. After this initial incubation, the lingual epithelium was peeled and incubated in Ca^2+^-free Tyrode’s solution for 26 min. The taste receptor cells were aspirated and plated on coverslips coated with Celltak (Corning Inc., Corning, NY).

### Live cell calcium imaging

All measurements of intracellular calcium were performed in isolated taste receptor cells that were not in contact with other taste cells as done in previous studies (Hacker et al., 2008; Dutta Banik et al., 2020). Cells were loaded for 20 minutes at room temperature (RT) with 2 μM Fura2-AM (ThermoFisher Scientific, Eugene, OR) containing Pluronic F-127 (ThermoFisher Scientific). Loaded cells were then washed in Tyrode’s solution under constant perfusion for 20min. Cells were visualized using an Olympus IX73 microscope with a 40X oil immersion lens and images were captured with a Hamamatsu ORCA-03G camera (Hamamatsu Photonics K.K., SZK Japan). Excitation wavelengths of 340nm and 380nm were used with an emission wavelength of 510nm. During experiments, cells were kept under constant perfusion using a gravity flow perfusion system (Automate Scientific, San Francisco, CA). Stimuli and pharmacological agents were applied using the gravity flow perfusion system. Images were collected every 4s using Imaging Workbench 6.0 (Indec Biosystems, Santa Clara, CA). Experiments were graphed and analyzed using Origin 2016 software (OriginLab Corporation, Northampton, MA).

Data from cells were analyzed if the cell had a stable Ca^2+^ baseline within the range of 65 nM and 200 nM. An evoked response was defined as measurable if the increase in fluorescence was at least 2 SD above a stable baseline and was reversible during the following washout period.

### Dual live cell imaging

We used two different dual live cell imaging techniques in this study. Dual calcium and sodium imaging was performed as previously described (Dutta Banik et al., 2018). Briefly, isolated cells were simultaneously loaded with Fura 2-AM (ThermoFisher) and Asante NaTrium-2 (TEFLabs, Inc., Austin, TX). For the dual calcium and membrane potential imaging, cells were simultaneously loaded with Fura 2-AM and DiBAC4(3) (ThermoFisher Scientific). In both sets of experiments, cells were excited at 340 nm, 380 nm, and 488 nm excitation wavelengths. The dual live cell images were captured every 4 s using a multiedge dichroic beam-splitter that captures emissions at both 510 nm and 540 nm using Imaging Workbench (Indec Biosystems). Experiments were graphed and analyzed using Origin software.

### Solutions

All chemicals were purchased from Sigma Aldrich unless otherwise noted. Tyrode’s solution contained 140 mM NaCl, 5 mM KCl, 3 mM CaCl_2_, 1 mM MgCl_2_, 10 mM HEPES, 10 mM glucose, and 1 mM pyruvate, pH 7.4. Ca^2+^-free Tyrode’s contained 140 mM NaCl, 5 mM KCl, 2.7 mM BAPTA, 2 mM EGTA, 10 mM HEPES, 10 mM glucose, 1 mM pyruvate, pH 7.4. HiK solution contained 50 mM KCl, 90 mM NaCl, 3 mM CaCl_2_, 1 mM MgCl_2_, 10 mM HEPES, 10 mM glucose, 1 mM pyruvate, pH 7.4. 100 mM HiK solution contained 100 mM KCl, 40 mM NaCl, 3 mM CaCl_2_, 1 mM MgCl_2_, 10 mM HEPES, 10 mM glucose, 1 mM pyruvate, pH 7.4. The taste mix contained 5 mM denatonium (bitter), 5 mM sucralose (sweet) and 5 mM MPG (umami) dissolved in normal Tyrode’s solution. Individual stimuli were also applied at the following concentrations: denatonium (5 mM), sucralose (10 mM) and mono-potassium glutamate (MPG) (20 mM). Salt (250 mM sodium chloride, NaCl) and sour (50mM citric acid, CA, pH4) stimuli were tested using the previously described protocol (Lewandowski et al., 2016; Dutta Banik et al., 2018). Inhibitor concentrations are indicated in parentheses: 9-Phenanthrol (50 μM), thapsigargin (2 μM), nickel chloride (200 μM), and cadmium (200 μM). The following inhibitors were from Tocris Bioscience (Minneapolis, MN): nimodipine (10 μM), *ϖ*-conotoxin GVIA (800 nM), *ϖ*-agatoxin IVA (300 nM), efonidipine (20 μM), and ryanodine (20 μM). All inhibitors were dissolved in normal Tyrode’s solution.

### Statistics

Statistical comparisons were made using either Student’s t-test or one-way ANOVAs with a Bonferroni’s and Tukey’s *post hoc* analysis using Origin 2016 software (OriginLab Corporation). For all analyses, a significance level of P < 0.05 was used. All graphs display individual data points, averages, and standard errors of the mean.

## Results

### TRPM4 functions downstream of PLCβ3 signaling pathway in broadly responsive (BR) Type III taste cells

**FIGURE 1:**
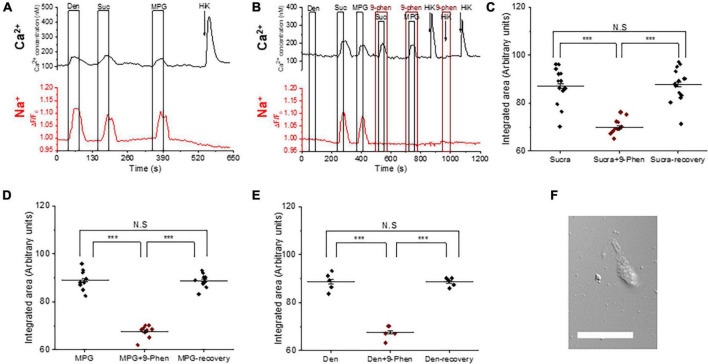
TRPM4 is solely responsible for the taste-evoked sodium responses in BR Type III cells. **(A)** Representative dual Ca^2+^ (black line) and Na^+^ (red line) imaging traces from BR cells showing cytosolic Ca^2+^ and Na^+^ responses to multiple taste stimuli: bitter (5 mM denatonium), sweet (10 mM sucralose), and umami (20 mM MPG). **(B)** Taste-evoked Na^+^ responses were eliminated by 9-phenanthrol, a selective TRPM4 inhibitor, in BR cells. The magnitudes (integrated area) of the taste-evoked Ca^2+^ responses were significantly reduced by 9-phenanthrol in BR cells for different stimuli: **(C)** sweet (*F*_(2,39)_ = 33.41, *p* = 0.0000000035, *n* = 14 cells from 5 mice), **(D)** umami (*F*_(2,24)_ = 120.83, *p* = 0.0000000000003, *n* = 9 cells from 5 mice), and **(E)** bitter (*F*_(2,12)_ = 85.43, *p* = 0.00000008, *n* = 5 cells from 3 mice) (***P < 0.001; N.S = Non-significant). **(F)** A DIC image of a representative taste receptor cell. Scale bar = 20 μM.

To determine if TRPM4 is downstream of the PLCβ3 signaling pathway in BR taste cells, we performed dual sodium-calcium live cell imaging. Live cell imaging has the advantage that we can collect data from a much larger population of cells than would be feasible using other approaches. This is important since we cannot preselect which cells will respond to a particular stimulus. Therefore, this approach allows us to perform more quantitative analyses. BR cells are identified by their calcium responses to bitter, sweet and/or umami stimuli and 50 mM KCl. We and others have previously applied 50mM KCl to identify the presence of VGCCs and functionally identify Type III taste cells (Richter et al., 2003; Hacker et al., 2008; Huang et al., 2008, 2011; Lewandowski et al., 2016; Sukumaran et al., 2017; Dutta Banik et al., 2018, 2020). We found that BR cells display simultaneous taste-evoked calcium and sodium responses for bitter, sweet, and umami stimuli (representative trace is shown Figure 1A). Consistent with our previous study (Dutta Banik et al., 2018), membrane depolarization with 50 mM KCl evoked a calcium signal but did not generate a measurable sodium signal in our experimental setup. This is because our live cell imaging experiments measure global changes in cytosolic sodium while the membrane depolarization needed to activate voltage gated channels is more localized. Therefore, while depolarization is expected to activate voltage-gated sodium channels, we will not detect those localized changes in sodium.

We used 9-phenanthrol to block TRPM4 in in our experiments since it is the only TRPM4 inhibitor currently available. While 9-phenanthrol is widely used to block TRPM4 activity, studies in other systems have reported that 9-phenanthrol can affect other channels in addition to TRPM4 (Burris et al., 2015; Veress et al., 2018). Most of these channels do not appear to be expressed in taste cells (Sukumaran et al., 2017) and our earlier study found that 9-phenanthrol selectively inhibits TRPM4 activity in taste cells (Dutta Banik et al., 2018). The taste-evoked sodium responses were abolished when 9-phenanthrol, the TRPM4 inhibitor, was applied (Figure 1B and Supplementary Figures 1A–C). These data suggest that TRPM4 is responsible for the bitter, sweet, or umami stimuli-evoked sodium responses in the BR cells and likely functions downstream of the PLCβ3 signaling pathway. Interestingly, inhibiting TRPM4 also reduced the magnitudes (integrated area) of the taste-evoked calcium signals (Figures 1B–E) but did not affect the amplitudes of the responses (Supplementary Figures 1D–F). A DIC image of an isolated taste receptor cell is shown in Figure 1F. To confirm the effects of TRPM4 activity on taste-evoked signaling in BR cells, we applied a taste mix (consisting of 5 mM denatonium, 5 mM sucralose, and 5 mM MPG) and 50 mM KCl to identify BR taste cells in WT and TRPM4-KO mice. Using this approach, we found that the taste-evoked sodium responses were absent in the BR cells of the TRPM4-KO mouse compared to responses from WT (Supplementary Figure 2).

We also evaluated the role of TRPM4 in sour and salt detection. Type III cells detect sour and salty stimuli through ionotropic receptors that depolarize the cell to activate VGCCs and cause neurotransmitter release (Huang et al., 2008; Kataoka et al., 2008; Chang et al., 2010; Oka et al., 2013; Lewandowski et al., 2016; Ye et al., 2016). We previously identified three populations of sour sensitive Type III cells: (1) responds to sour only, (2) responds to sour as well as bitter, sweet and/or umami, and (3) responds to sour and salt but not to bitter, sweet, and umami stimuli (Dutta Banik et al., 2020). We found that inhibiting TRPM4 significantly reduced the sour-evoked calcium signals by citric acid (CA) in BR Type III cells (Supplementary Figures 3A, B). Conversely, in the Type III cells that did not respond to the taste mix (non-BR Type III cells), TRPM4 inhibition did not affect the salt and sour responses (Supplementary Figures 3C–E). Since the biggest effect of inhibiting TRPM4 activity was in the bitter, sweet, and/or umami signaling in BR cells, we chose to focus our studies on identifying its role in these signaling events. Future studies will characterize the role of TRPM4 in sour taste.

**FIGURE 2:**
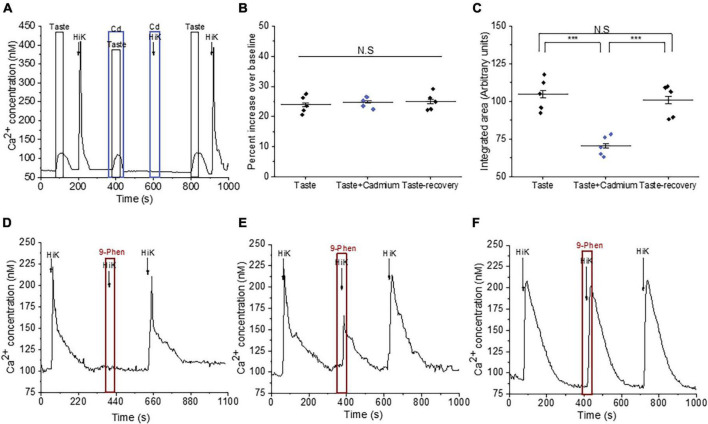
TRPM4 modulates the activity of VGCCs in Type III taste cells. **(A)** A representative calcium imaging trace showing cadmium (200 μM) reduced the taste-evoked calcium responses and abolished the calcium influx signals generated by 50 mM KCl. **(B,C)** Cadmium, a general VGCC blocker, did not affect the amplitudes of the taste-evoked calcium responses (*F*_(2,12)_ = 0.23; *p* = 0.79) but significantly reduced the magnitudes (integrated area) (*F*_(2,12)_ = 18.99, *p* = 0.00019, *n* = 5 cells from 5 mice). **(D)** Inhibition of TRPM4 abolished the calcium influx signals in some Type III cells (*n* = 15 cells from 3 mice), while 9-phenanthrol partially inhibited calcium influx in other cells (**E**; *n* = 9 cells from 3 mice). **(F)** In other Type III cells, TRPM4 inhibition did not affect the calcium influx signals (*n* = 24 cells from 7 mice) (***P < 0.001; N.S = Non-significant).

We followed up on our observation that TRPM4 activity affects the evoked calcium responses in BR cells. Because TRPM4 is a monovalent cation selective channel, it does not directly contribute to the taste-evoked calcium signals but is likely modulating the activity of other channels that can pass calcium. BR cells are a subset of Type III cells that express VGCCs (Dutta Banik et al., 2020) and TRPM4 has been shown to modulate VGCC activity in other cell types (Earley et al., 2004, 2007; Gonzales and Earley, 2012; Menigoz et al., 2016), so it is possible that they regulate VGCC activity in the BR cells as well. To test this idea, we measured the calcium responses to a taste mix (as previously defined) in the presence and absence of cadmium (200 μM), which blocks all VGCC channels (Figure 2A). Cadmium did not affect the initial amplitudes of the taste-evoked responses, but significantly reduced the overall magnitudes of the evoked calcium signals (Figures 2B, C), which was similar to the effect of TRPM4 inhibition on the responses (Figure 1). We concluded that calcium influx through VGCCs likely contributes to these taste-evoked responses and that their activity may be regulated by TRPM4 during this signaling event.

To establish if TRPM4 directly regulates the calcium influx through VGCCs, we depolarized the cells with 50 mM KCl in conjunction with 9-phenanthrol and found that TRPM4 inhibition completely blocked calcium influx in a subset of Type III cells (Figure 2D and Supplementary Figure 4A), while partially blocking the calcium influx signals in other cells (Figure 2E and Supplementary Figure 4B). TRPM4 activity did not affect the calcium influx signals in a third group of cells (Figure 2F and Supplementary Figure 4C), which suggests that TRPM4 selectively regulates a subset of VGCCs in Type III taste cells.

### TRPM4 does not interact with P/Q, N, and T-type VGCCs in BR Type III cells

To determine if TRPM4 regulates specific VGCC isoforms, we measured the effect of inhibiting TRPM4 with different VGCC antagonists. We reasoned that if TRPM4 associates with a specific VGCC isoform, then inhibiting either one or both channels would cause a comparable inhibition of the calcium influx signals. However, if their activities were independent, then blocking both channels would increase the overall inhibition of the response. Since Type III taste cells express N, P/Q, T, and L-type but not R-type VGCCs (Roberts et al., 2009; Rebello et al., 2013), we tested for potential interactions between TRPM4 and these VGCC isoforms. Data are reported as percent inhibition of the control responses.

**FIGURE 3:**
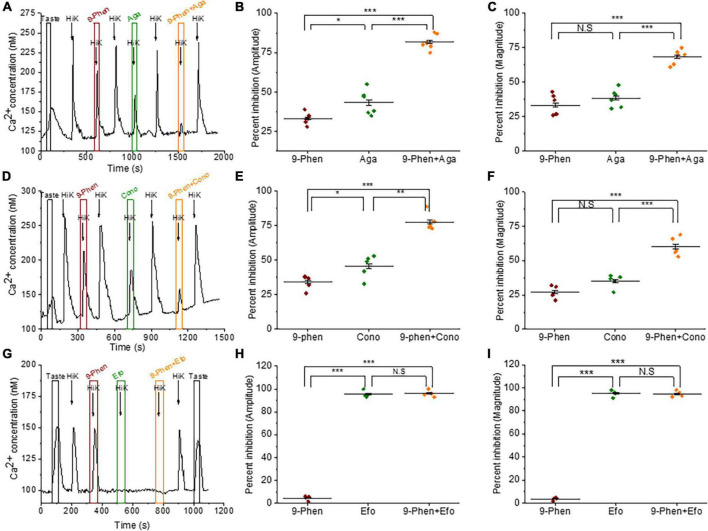
TRPM4 does not contribute to calcium signals due to P/Q, N, and T-type VGCCs. **(A)** A representative calcium imaging trace showing inhibition of the 50 mM KCl responses by 9-phenanthrol (50 μM), ω-agatoxin IVA (300 nM), and ω-agatoxin IVA + 9-phenanthrol in BR cells. Analysis of the 50 mM KCl signals revealed that co-application of ω-agatoxin IVA and 9-phenanthrol significantly increased the level of inhibition for both **(B)** signal amplitudes (*F*_(2,15)_ = 117.40, *p* = 0.00000000069) and **(C)** magnitudes (*F*_(2,15)_ = 50.68, *p* = 0.00000021) compared to inhibiting either channel alone (*n* = 6 cells from 3 mice). **(D)** A representative calcium imaging trace showing inhibition of the 50 mM KCl responses by 9-phenanthrol, ω-conotoxin GVIA (800 nM), and ω-conotoxin GVIA + 9-phenanthrol in BR cells. The level of inhibition for both **(E)** the amplitudes (*F*_(2,12)_ = 56.10, *p* = 0.00000081) and **(F)** the magnitudes (*F*_(2,12)_ = 49.08, *p* = 0.0000017) of the responses significantly increased with the co-application of ω-conotoxin GVIA and 9-phenanthrol compared to the inhibition of either channel alone (*n* = 5 cells from 3 mice). **(G)** A representative calcium imaging trace of the calcium responses to 30 mM KCl in BR cells. **(H,I)** This signal was not blocked by 9-phenanthrol, but was completely inhibited by ephonidipine (20 μM), a T-type VGCC blocker in BR cells (*n* = 4 cells from 3 mice) (*P < 0.05; **P < 0.01; ***P < 0.001; N.S = Not significant).

We used *ϖ*-agatoxin IVA to selectively block P/Q-type VGCCs (Ca_*v*_2.1) in the presence or absence of 9-phenanthrol. Simultaneous inhibition of TRPM4 and P/Q-type VGCCs during cell depolarization further inhibited both the amplitudes and magnitudes of the calcium influx signals compared to blocking the channels separately (Figures 3A–C). These data indicate that TRPM4 and P/Q-type VGCCs independently contribute to the calcium influx signal in BR Type III cells. When we repeated this experiment with 9-phenanthrol and *ϖ*-conotoxin GVIA (a specific inhibitor of N-type VGCCs) in BR Type III cells, we got similar results (Figures 3D–F).

We also tested for a potential interaction between TRPM4 and T-type VGCCs (Ca_*v*_3.x). Since T-type VGCCs are low voltage activated channels (LVA), we used 30 mM KCl to depolarize the cells to ∼-40 mV, which is close to the activation voltage for T-type channels (Perez-Reyes, 2003). These signals were completely blocked by efonidipine, a T-type VGCC inhibitor (Figure 3G) as well as NiCl_2_, another T-type channel inhibitor (Supplementary Figure 5). Inhibiting TRPM4 did not affect this calcium signal (Figures 3H, I), and we concluded that TRPM4 and T-type VGCCs do not interact in BR Type III cells.

### TRPM4 functionally interacts with L-type VGCCs in BR Type III cells

**FIGURE 4:**
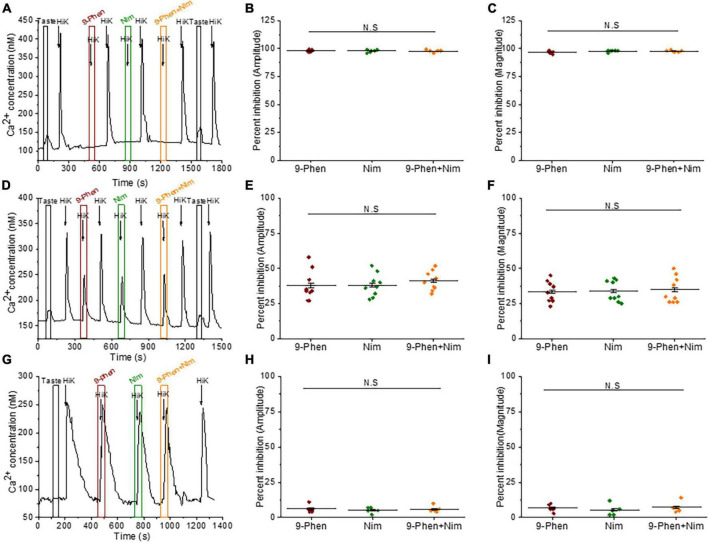
TRPM4 regulates L-type VGCC activity in BR cells. **(A–C)** In some BR cells, the calcium signals due to 50 mM KCl were abolished by both 9-phenanthrol (50 μM) and nimodipine (10 μM) (amplitude; *F*_(2,15)_ = 0.32, *p* = 0.73) (magnitude; *F*_(2,15)_ = 2.14, *p* = 0.15, *n* = 6 cells from 3 mice). **(D)** In other BR cells, calcium influx was partially reduced when either TRPM4 or L-type VGCCs were individually inhibited as well as when they were co-inhibited. **(E)** The percent inhibition of the peak amplitudes (*F*_(2,24)_ = 0.36, *p* = 0.70) and **(F)** magnitudes (*F*_(2,24)_ = 0.06, *p* = 0.94) of the calcium responses were comparable when either 9-phenanthrol, nimodipine or 9-phenanthrol + nimodipine were applied (*n* = 9 cells from 5 mice). In non-BR Type III cells, inhibiting TRPM4 or L-type VGCC activity did not affect the calcium signals due to 50 mM KCl. **(G)** A representative trace is shown. Neither the peak amplitudes (**H**, *F*_(2,12)_ = 0.23, *p* = 0.79) or peak magnitudes (**I,** *F*_(2,12)_ = 0.40, *p* = 0.68) of the calcium signals were affected by either inhibitor (*n* = 5 cells from 3 mice) (N.S = Not significant).

The only other identified VGCC isoform in Type III cells is the L-type channel (Ca_*v*_1.x) (Roberts et al., 2009; Rebello et al., 2013), which can be modulated by TRPM4 in other systems (Welsh et al., 2002; Earley et al., 2004, 2007; Gonzales and Earley, 2012; Menigoz et al., 2016). We found that inhibition of either TRPM4 or L-type VGCCs (with 10 μM nimodipine) completely blocked the calcium signals in some BR cells (Figures 4A–C). In other cells, inhibiting either TRPM4 or L-type VGCCs caused comparable reductions in the calcium influx and concurrent blocking of TRPM4 and L-type VGCCs did not cause any further inhibition (Figures 4D–F). The calcium influx signals due to 50mM KCl from non-BR Type III cells were not affected by inhibiting either TRPM4 or L-type VGCCs (Figures 4G–I). Based on these data, we concluded that TRPM4 selectively regulates L-type VGCCs and this interaction is primarily found in BR cells.

### Calcium influx through L-type VGCC contributes to the taste-evoked calcium responses in BR Type III cells

**FIGURE 5:**
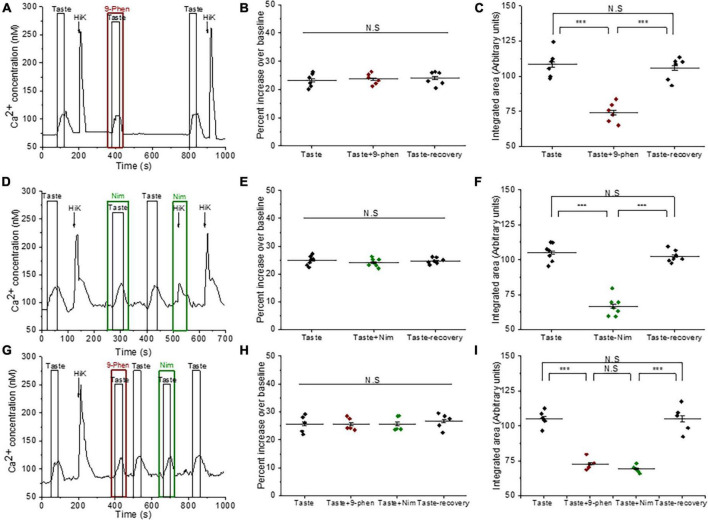
The calcium influx by L-type VGCCs contributes to taste-evoked calcium responses in BR cells. **(A)** A representative calcium imaging trace showing inhibition of a taste-evoked calcium response by 9-phenanthrol (50 μM). **(B)** Inhibiting TRPM4 did not significantly affect the amplitudes of the taste-evoked calcium responses (*F*_(2,15)_ = 0.16, *p* = 0.85), but caused a significant reduction in the signal magnitudes (**C**, *F*_(2,15)_ = 32.07, *p* = 0.0000038, *n* = 6 cells from 3 mice). **(D)** A representative calcium imaging trace showing inhibition of taste-evoked calcium responses by nimodipine (10 μM). **(E)** Nimodipine did not inhibit the amplitudes of the taste-evoked responses (*F*_(2,18)_ = 0.48, *p* = 0.62), but significantly reduced the signal magnitudes (**F**, *F*_(2,18)_ = 89.55, *p* = 0.00000000044, *n* = 7 cells from 4 mice). **(G–I)** Similar results were observed when TRPM4 and L-type channels were independently blocked in the same BR cells (amplitude; *F*_(3,16)_ = 0.17, *p* = 0.91) (magnitude; *F*_(3,16)_ = 49.76, *p* = 0.000000025, *n* = 5 cells from 3 mice) (***P < 0.001; N.S = Not significant).

So far, our data indicate that TRPM4 works downstream of the PLCβ3 signaling pathway and selectively regulates L-type VGCCs in BR Type III cells. Since cadmium reduced the taste-evoked calcium signals in these cells (Figures 2A, C), we tested the hypothesis that these two signaling events are related. Inhibiting TRPM4 did not affect the initial amplitudes of the taste-evoked responses, but significantly reduced the magnitudes of the signals (Figures 1B–E, 5A-C). Similarly, inhibiting L-type VGCCs significantly reduced the magnitudes, but not the amplitudes, of the taste-evoked responses (Figures 5D–F). Inhibiting both channels caused comparable reductions in the size of the taste signals (Figures 5G–I). Control experiments for the other VGCC isoforms determined that these effects were specific to L-type channels (Supplementary Figure 6). We concluded that TRPM4 likely activates L-type VGCCs which contributes to the taste-evoked calcium responses in BR cells.

### TRPM4 modulates the activity of VGCCs by depolarizing the cell membrane

**FIGURE 6:**
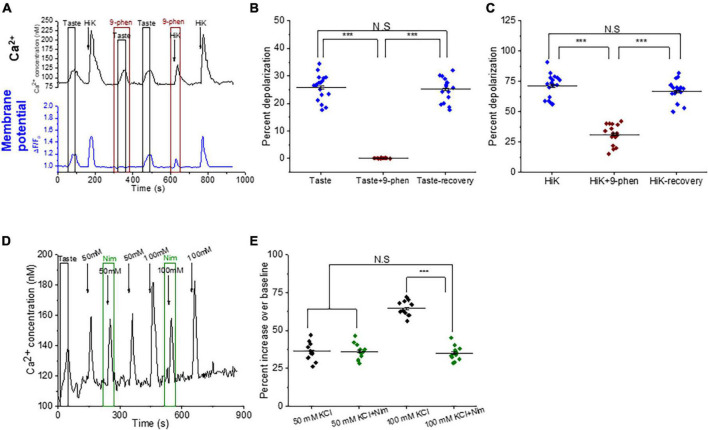
TRPM4-mediated depolarization is necessary to activate L-type VGCCs in BR cells. **(A)** Representative dual Ca^2+^ (black line) and membrane potential (blue line) imaging traces from BR cells showing simultaneous taste and 50 mM KCl-evoked calcium signals and membrane depolarization. Inhibiting TRPM4 abolished the taste-evoked depolarization (**B**, *F*_(2,51)_ = 299.68, *p* = 0), while the KCl-induced depolarization was significantly reduced (**C**, *F*_(2,51)=_109.90, *p* = 0, *n* = 18 cells from 6 mice). **(D)** A representative calcium imaging trace from the TRPM4-KO mice. **(E)** In the TRPM4-KO cell, 50 mM KCl generated a small calcium influx that was not inhibited by nimodipine (10 μM). When 100 mM KCl was applied, the calcium influx signals increased and were then partially inhibited by nimodipine (10 μM) (*F*_(3,40)_ = 77.71, *p* = 0, *n* = 11 cells from 4 mice) (***P < 0.001; N.S = Not significant).

In other cell types, TRPM4 depolarizes the membrane sufficiently to activate L-type VGCCs (Launay et al., 2002, 2004; Earley et al., 2004; Guinamard et al., 2015; Menigoz et al., 2016). To determine if this occurs in taste cells, we used dual calcium-membrane potential imaging to measure simultaneous changes in intracellular calcium and membrane potential. Applying either taste mix or 50 mM KCl depolarized the taste cell (Figure 6A). Inhibiting TRPM4 abolished the taste-induced depolarization (Figures 6A, B) and significantly reduced the depolarization caused by 50 mM KCl (Figures 6A, C). These data confirm that TRPM4 is depolarizing the membrane in response to taste stimuli which we predict is affecting VGCC activity.

Since TRPM4 activity significantly increases membrane depolarization, we did a follow up study with TRPM4-KO mice to determine if TRPM4-mediated depolarization is needed for L-type VGCC activation in BR cells. In the TRPM4-KO mice, calcium responses to 50 mM KCl in BR cells were no longer inhibited by either 9-phenanthrol or nimodipine (Supplementary Figures 7A–C), indicating these depolarization-induced calcium signals were not mediated by L-type VGCCs. This contrasts with the data in WT mice which demonstrated that inhibiting L-type VGCCs significantly reduced the taste-evoked calcium signals (Figures 4A, D). There are two possible explanations for this finding. It may be that L-type VGCCs are absent in TRPM4-KO mice due to genetic remodeling in these transgenic mice. The second possibility is that L-type VGCCs are still expressed in BR cells but are not activated due to the absence TRPM4 activity. We reasoned that if TRPM4-mediated depolarization is normally required to activate L-type VGCCs, then a stronger depolarization could compensate for the absence of TRPM4 activity. To test this hypothesis, we increased the cell depolarization (100 mM KCl) and recorded a larger calcium influx, which was then partially inhibited by nimodipine (Figure 6D). Thus, L-type VGCCs are expressed and can be activated by a stronger depolarization in these cells (Figure 6E). Based on these data, we concluded that TRPM4-mediated membrane depolarization is required to appropriately activate L-type VGCCs in BR cells.

### Calcium-induced calcium release through ryanodine receptor amplifies the taste-evoked signaling in BR Type III cells

**FIGURE 7:**
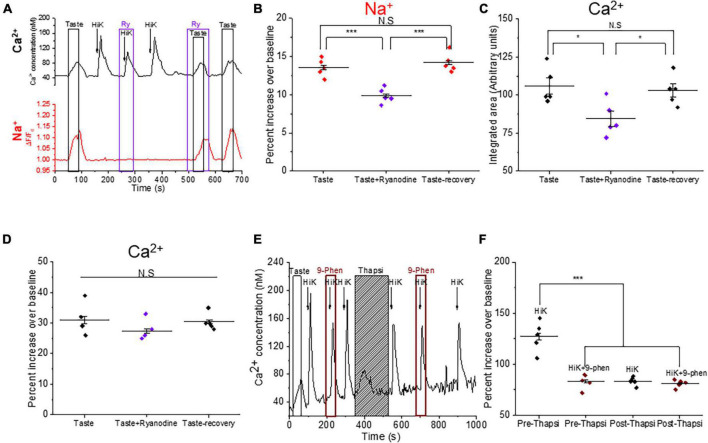
Calcium-induced calcium release (CICR) signaling via ryanodine receptors contributes to the taste-evoked signaling in BR cells. **(A)** Representative dual Ca^2+^ (black line) and Na^+^ (red line) imaging traces from BR cells showing ryanodine (20 μM) inhibited the taste-evoked sodium and calcium responses. **(B)** The taste-evoked sodium responses were significantly reduced by ryanodine (20 μM) ((*F*_(2,12)_ = 20.99, *p* = 0.00012, *n* = 5 cells from 3 mice). Ryanodine significantly reduced the magnitudes of the taste-evoked calcium signals (**C**, *F*_(2,12)_ = 5.66, *p* = 0.02), but did not affect the amplitudes of the taste-evoked calcium responses (**D**, *F*_(2,12)_ = 1.29, *p* = 0.31, *n* = 5 cells from 3 mice). **(E)** A representative calcium imaging trace in BR cell showing CICR signaling affects TRPM4 activity. **(F)** 9-phenanthrol partially inhibited the calcium influx signals generated by 50 mM KCl in some BR cells. After store depletion by thapsigargin, peak amplitudes of the calcium responses due to 50 mM KCl were comparable to the peak amplitudes of the calcium responses during application of 9-phenanthrol. TRPM4 inhibition no longer affected the calcium signals after store depletion (*F*_(3,16)_ = 33.15, *p* = 0.00000043, *n* = 5 cells from 3 mice). (*P < 0.05; ***P < 0.001).

We previously reported that L-type VGCCs are functionally coupled with ryanodine receptors (RyRs) in Type III taste cells to generate a calcium-induced calcium release signal (CICR) (Rebello and Medler, 2010; Rebello et al., 2013). Since a CICR signal via RyRs has been shown to potentiate TRPM4 activation in myocytes (Morita et al., 2007), we asked whether CICR signaling also modulates TRPM4 activity in BR cells. Using dual calcium-sodium imaging, we found that inhibiting RyRs significantly reduced the taste-evoked sodium responses (Figures 7A, B). Inhibition of RyRs also significantly reduced the magnitudes of the taste-evoked calcium responses but did not affect the amplitudes of these signals (Figures 7C, D). Thus, RyRs are activated during taste-evoked signaling in BR cells and contribute to the activation of TRPM4.

To further explore the relationship between TRPM4 and CICR signals, we depleted internal calcium stores with the irreversible SERCA inhibitor, thapsigargin (2 μM) and measured the effect of TRPM4 activity on the calcium influx signal. We postulated that CICR signaling may activate TRPM4 which would then activate more VGCCs. If this feedback event occurs in BR cells, then depleting internal stores would result in a smaller calcium signal in response to 50 mM KCl. We first confirmed that blocking TRPM4 inhibits calcium influx in these cells and then depleted the internal calcium stores. After store depletion, 50 mM KCl produced a smaller calcium signal that was comparable to the calcium influx signal when TRPM4 was inhibited prior to store depletion (Figure 7E). After internal calcium stores were depleted, the calcium influx signals were no longer affected by the TRPM4 antagonist (Figure 7F). These data indicate that calcium release from internal stores activates TRPM4, which enhances the VGCC activity in the BR cells.

## Discussion

**FIGURE 8:**
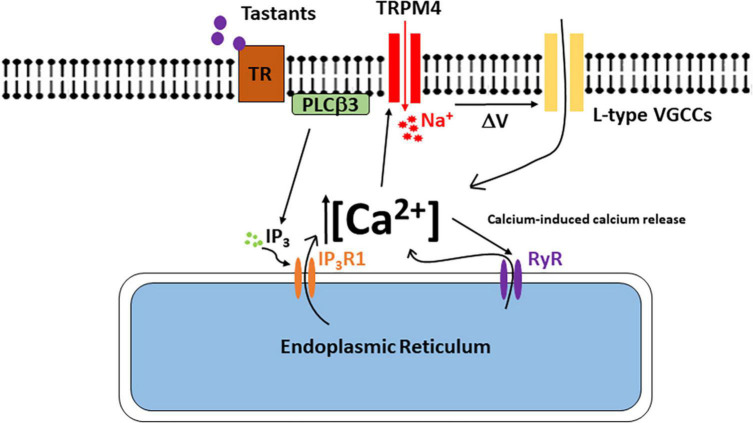
The model of signaling events in the BR Type III cells. Bitter, sweet, and/or umami stimuli bind to the taste receptor and activate the PLCβ3 signaling pathway. Activation of this signaling pathway causes calcium release from the internal stores which activates TRPM4. Na^+^ ions enter the cell through TRPM4 and depolarizes the cell to activate L-type VGCCs either directly or via changes in action potential activity. Ca^2+^ influx though L-type VGCCs also generates a CICR signal via ryanodine receptors. This CICR signal enhances TRPM4 activation to further depolarize the cell and activate additional L-type VGCCs.

This study has identified TRPM4 as an important downstream target in bitter, sweet, and umami signaling in the BR Type III cells (see model in Figure 8). Taste-evoked activation of the PLCβ3 signaling pathway causes an initial calcium release from internal stores that activates TRPM4 which depolarizes the membrane and triggers the opening of L-type VGCCs. Since inhibiting either TRPM4 or L-type VGCCs did not affect the amplitudes of the taste-evoked calcium signals, we conclude that the initiation of the taste signal is due solely to calcium release from the internal stores as the result of activating the PLCβ3/IP_3_R1 signaling pathway. TRPM4 activation and the subsequent L-type VGCC activity occur downstream of this initial calcium signal and contribute to the overall taste-evoked calcium signal since the magnitudes of the taste responses in the BR cells were significantly reduced when these channels were inhibited.

Type III taste cells have conventional chemical synapses that rely on the activation of voltage-gated calcium channels through the firing of action potentials to cause neurotransmitter release (Vandenbeuch and Kinnamon, 2009). We have previously shown that BR cells, which are a subpopulation of Type III cells, produce calcium signals in response to bitter, sweet, and umami stimuli using a PLCβ3 signaling pathway (Dutta Banik et al., 2020). However, the underlying signaling mechanisms which connect this taste-evoked calcium signal to neurotransmitter release were unknown. Our data suggest that TRPM4 is the link between these two signaling events.

TRPM4 has well-established and wide ranging roles in regulating membrane potential (Earley et al., 2004; Gonzales et al., 2010; Guinamard et al., 2015; Kurland et al., 2016; Menigoz et al., 2016; Simard and Gerzanich, 2018; Stokum et al., 2018; Ozhathil et al., 2021; Riquelme et al., 2021; Simard et al., 2021). In CA1 hippocampal neurons, loss of TRPM4 does not affect the intrinsic properties of the action potential but reduces the number of action potentials fired during depolarization (Menigoz et al., 2016). In atrial cardiomyocytes, TRPM4 directly influences the properties of the action potentials (Simard et al., 2013), while TRPM4 contributes to the resting membrane potential in smooth muscle cells (Gonzales et al., 2010). Earlier studies have shown that TRPM4-dependent depolarization can modulate VGCC activity, primarily L-type VGCCs, and that this interaction is important for a variety of physiological processes (Earley et al., 2004, 2007; Takezawa et al., 2006; Gonzales et al., 2010; Rubi et al., 2013). We have now shown that this TRPM4-L-type VGCC connection is also present in BR taste cells, where it likely affects neurotransmitter release.

To determine if TRPM4 activity is required to appropriately activate L-type VGCCs, we measured the L-type VGCC response in the absence of TRPM4. Using TRPM4-KO mice, we found that L-type VGCCs are not significantly activated in BR cells when TRPM4 is absent. Our finding is consistent with a study in hippocampal neurons which reported that TRPM4 is required to activate L-type VGCCs and maintain long term potentiation (LTP) (Menigoz et al., 2016). In these neurons, induction of LTP activates TRPM4 which leads to calcium influx through L-type VGCCs. When TRPM4 was absent, LTP was still initiated, but could no longer be maintained. However, LTP maintenance was rescued by a stronger depolarization that compensated for the loss of TRPM4 and activated the L-type VGCCs. Similarly, we found that a stronger depolarization activated L-type VGCCs in the BR cells when TRPM4 was absent. These data suggest that TRPM4 is normally required to appropriately activate L-type VGCCs in BR cells. Since we did not test the effects of TRPM4 on action potential activity in BR Type III taste cells directly, we cannot conclude if the effect of TRPM4 on L-type VGCCs is due to altered action potential firing or more generalized changes to the membrane potential.

Interestingly, TRPM4 activity is modulated by calcium-induced calcium release (CICR) in BR cells. We previously reported that ryanodine receptors and L-type VGCCs are functionally associated in a subset of Type III cells to produce CICR (Rebello et al., 2013). Our current data suggest that this CICR enhances cell depolarization by activating TRPM4 which then triggers the further opening of L-type VGCCs to amplify the initial calcium signal. This type of signal amplification mechanism has been shown to be important for excitation-contraction coupling in cardiac muscles as well as for neurotransmitter release in photoreceptor cells (Yamazawa et al., 1996; Krizaj et al., 1999; Coussin et al., 2000; Wang et al., 2001; Babai et al., 2010). Our data suggest that this CICR dependent signal amplification is also important in BR taste cells.

While it is generally thought that TRPM4 activity is dependent on calcium release from the internal stores (Launay et al., 2002; Murakami et al., 2003; Nilius et al., 2003; Earley et al., 2004), one study reported that TRPM4 is directly activated by calcium influx via the NMDA receptor (Menigoz et al., 2016). Still other studies have concluded that calcium release from stores and calcium influx work in tandem to activate TRPM4 (Gonzales et al., 2014; Tran et al., 2014). While we cannot rule out that TRPM4 is directly activated by calcium influx in BR cells, our data suggest that calcium influx generates a CICR signal which then activates TRPM4. This positive signaling loop between TRPM4, L-type VGCCs and ryanodine receptors appears to be needed to generate the appropriate cellular response to taste stimuli in these cells.

Overall, our data identifies TRPM4 as an important downstream effector of the PLCβ3/IP_3_R1 signaling pathway in BR Type III cells and serves as the link between the initial taste-evoked calcium release signal and the subsequent VGCC activity that is required for neurotransmitter release.

## Data availability statement

The raw data supporting the conclusions of this article will be made available by the authors, without undue reservation.

## Ethics statement

This animal study was reviewed and approved by University at Buffalo Institutional Animal Care and Use Committee.

## Author contributions

KM conceived the study. DDB and KM designed the research, performed the experiments, analyzed the data, and wrote the manuscript. Both authors contributed to the article and approved the submitted version.

## Supplementary material

Supplementary Figure 1 TRPM4 inhibition abolishes taste-evoked sodium responses in BR Type III cells. Application of 9-phenanthrol, the selective TRPM4 inhibitor, abolished the taste-evoked sodium responses for sweet (**A**, sucralose; *F*_(2,39)_ = 267.28, *p* = 0, *n* = 14 cells from 5 mice), umami (**B**, MPG; *F*_(2,24)_ = 326.47, *p* = 0, *n* = 9 cells from 5 mice), and bitter (**C**, denatonium; *F*_(2,12)_ = 336.04, *p* = 0, *n* = 5 cells from 3 mice). 9-phenanthrol did not affect the amplitudes of the taste-evoke calcium responses for sweet (**D**, sucralose; *F*_(2,39)_ = 0.008, *p* = 0.99, *n* = 14 cells from 5 mice), umami (**E**, MPG; *F*_(2,24)_ = 0.27, *p* = 0.77, *n* = 9 cells from 3 mice), and bitter (**F**, denatonium; *F*_(2,12)_ = 0.16, *p* = 0.86, *n* = 5 cells from 3 mice) (^***^*P* < 0.001; N.S = Not significant).

Supplementary Figure 2 BR cells lack taste-evoked sodium responses in TRPM4-KO mice. **(A)** BR cells in WT mice produce evoked cytosolic sodium and calcium increases when the taste mix is applied (n = 5 cells from 3 mice). **(B)** BR cells in TRPM4-KO mice fail to produce a cytosolic sodium increase to a taste mix, even though the taste-evoked calcium response is generated (*n* = 5 cells from 3 mice).

Supplementary Figure 3 TRPM4 partially regulates the sour responses in BR cells but has no effect on the sour and salty stimuli in other Type III cells. **(A)** A representative calcium imaging trace showing that citric acid evoked calcium signal was partially inhibited by 9-phenathrol. **(B)** Inhibition of TRPM4 significantly reduced the amplitudes of the calcium responses to citric acid in BR cells (F_(2,33)_ = 62.15, p = 0, n = 12 cells from 5 mice). **(C)** A representative calcium imaging trace showing TRPM4 did not affect the sour and salty responses in non-BR Type III cells. Application of 9-phenanthrol did not affect the calcium responses to salt (**D**, NaCl; *p* = 0.84, *n* = 7 cells from 3 mice) or sour (**E**, citric acid; *p* = 0.63, *n* = 7 cells from 3 mice) in the non-BR Type III cells (^***^*P* < 0.001; N.S = Not significant).

Supplementary Figure 4 Summary of the effects of 9-phenanthrol on VGCC activity in Type III cells. **(A)** Inhibition of TRPM4 abolished the calcium influx signals in some BR cells (*F*_(2,42)_ = 38.5, *p* = 0.00000000031, *n* = 15 cells from 3 mice), while 9-phenanthrol partially inhibited calcium influx in other BR cells (**B**, *F*_(2,24)_ = 12.9, *p* = 0.00015, *n* = 9 cells from 3 mice). **(C)** In other Type III cells, TRPM4 inhibition with 9-phenanthrol did not affect the calcium influx signals (*F*_(2,69)_ = 0.54, *p* = 0.947, *n* = 24 cells from 7 mice) (^***^*P* < 0.001; N.S = Non-significant).

Supplementary Figure 5 TRPM4 does not interact with T-type VGCCs in BR cells. A representative calcium imaging trace showing that the calcium response generated by 30 mM KCl was not inhibited by 9-phenanthrol, however, this signal was completely inhibited by nickel chloride, a T-type VGCC blocker (*n* = 5 cells from 3 mice).

Supplementary Figure 6 Calcium influx through P/Q, N, and T-type VGCCs does not contribute to taste-evoke calcium responses in BR cells. **(A,B)** Application of ω-agatoxin IVA (300 nM) did not affect the magnitudes of the taste-evoked calcium responses in BR cells (F_(2,12)_ = 0.17, p = 0.85, n = 5 cells from 3 mice). **(C,D)** Application of ω-conotoxin GVIA (800 nM) did not affect the magnitudes of the taste-evoked calcium responses in BR cells (*F*_(2,12)_ = 0.004, *p* = 0.99, *n* = 5 cells from 3 mice). **(E,F)** Application of ephonididpine (20 μM) also did not affect the magnitudes of the taste-evoked calcium responses in BR cells (F_(2,12)_ = 0.07, p = 0.94, n = 5 cells from 3 mice).

Supplementary Figure 7 BR cells are present in TRPM4-KO mice but 50mM KCl responses are no longer affected by inhibitors. **(A)** A representative calcium imaging trace of a BR taste cell from TRPM4-KO mice. In the TRPM4-KO BR cells, neither **(B)** the amplitudes nor **(C)** the magnitudes of the calcium signals generated by 50 mM KCl were inhibited by either 9-phenanthrol (50 μM) or nimodipine (10 μM) (amplitude; *F*_(3,12)_ = 1.38, *p* = 0.30; magnitude; *F*_(3,12)_ = 2.55, *p* = 0.10, *n* = 4 cells from 3 mice).
