# Supplementary figures and images for: Defining the role of TRPM4 in broadly responsive taste receptor cells
Source: Front Cell Neurosci. 2023 Mar 22;17:1148995. doi: 10.3389/fncel.2023.1148995 (PMC10073513; doi:10.3389/fncel.2023.1148995)

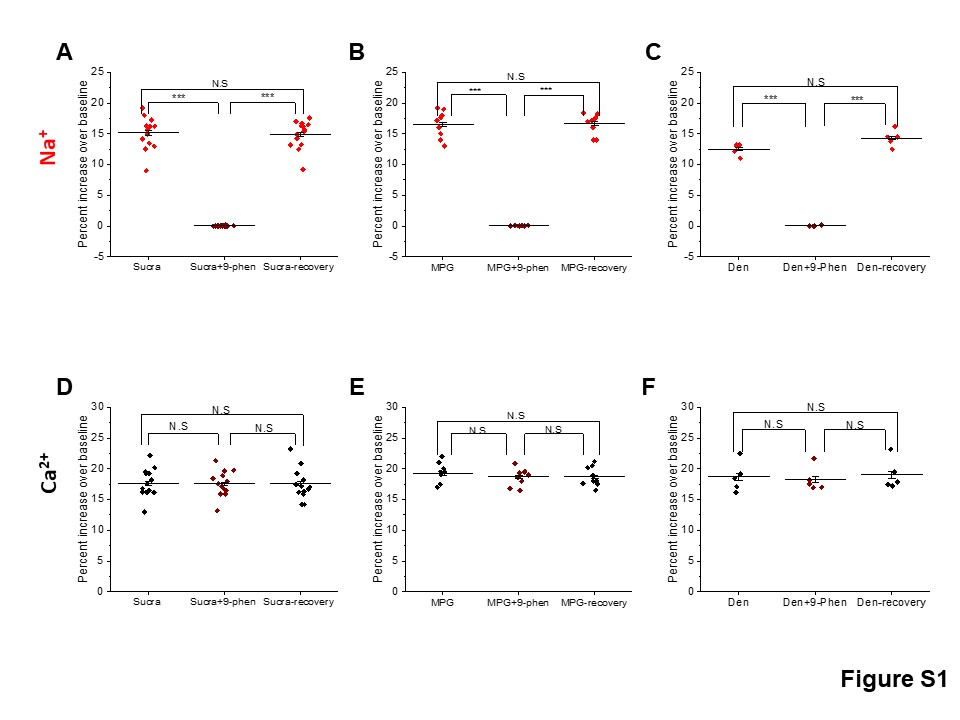

Supplement: Supplementary Figure 1 — TRPM4 inhibition abolishes taste-evoked sodium responses in BR Type III cells. Application of 9-phenanthrol, the selective TRPM4 inhibitor, abolished the taste-evoked sodium responses for sweet (A, sucralose; F(2,39) = 267.28, p = 0, n = 14 cells from 5 mice), umami (B, MPG; F(2,24) = 326.47, p = 0, n = 9 cells from 5 mice), and bitter (C, denatonium; F(2,12) = 336.04, p = 0, n = 5 cells from 3 mice). 9-phenanthrol did not affect the amplitudes of the taste-evoke calcium responses for sweet (D, sucralose; F(2,39) = 0.008, p = 0.99, n = 14 cells from 5 mice), umami (E, MPG; F(2,24) = 0.27, p = 0.77, n = 9 cells from 3 mice), and bitter (F, denatonium; F(2,12) = 0.16, p = 0.86, n = 5 cells from 3 mice) (***P < 0.001; N.S = Not significant). [file Image_1.jpeg]

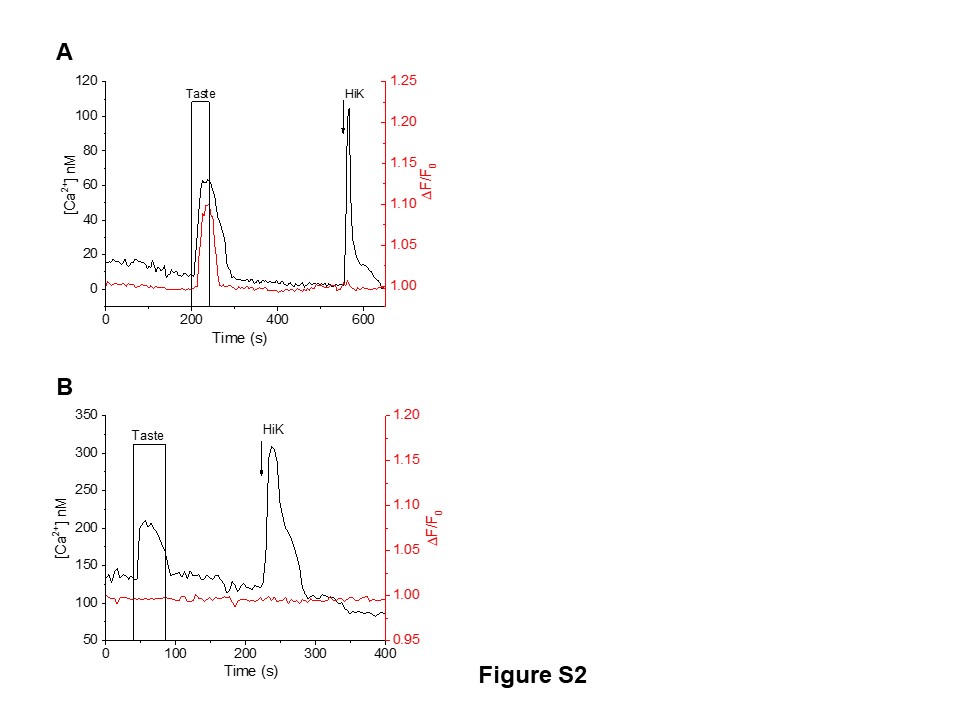

Supplement: Supplementary Figure 2 — BR cells lack taste-evoked sodium responses in TRPM4-KO mice. (A) BR cells in WT mice produce evoked cytosolic sodium and calcium increases when the taste mix is applied (n = 5 cells from 3 mice). (B) BR cells in TRPM4-KO mice fail to produce a cytosolic sodium increase to a taste mix, even though the taste-evoked calcium response is generated (n = 5 cells from 3 mice). [file Image_2.jpeg]

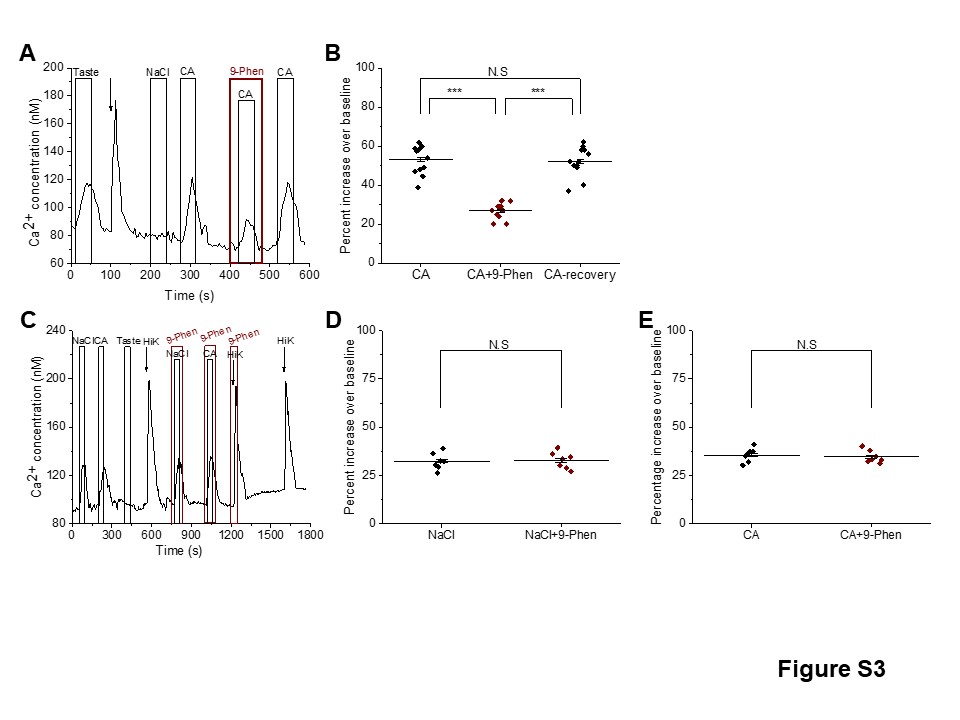

Supplement: Supplementary Figure 3 — TRPM4 partially regulates the sour responses in BR cells but has no effect on the sour and salty stimuli in other Type III cells. (A) A representative calcium imaging trace showing that citric acid evoked calcium signal was partially inhibited by 9-phenathrol. (B) Inhibition of TRPM4 significantly reduced the amplitudes of the calcium responses to citric acid in BR cells (F(2,33) = 62.15, p = 0, n = 12 cells from 5 mice). (C) A representative calcium imaging trace showing TRPM4 did not affect the sour and salty responses in non-BR Type III cells. Application of 9-phenanthrol did not affect the calcium responses to salt (D, NaCl; p = 0.84, n = 7 cells from 3 mice) or sour (E, citric acid; p = 0.63, n = 7 cells from 3 mice) in the non-BR Type III cells (***P < 0.001; N.S = Not significant). [file Image_3.jpeg]

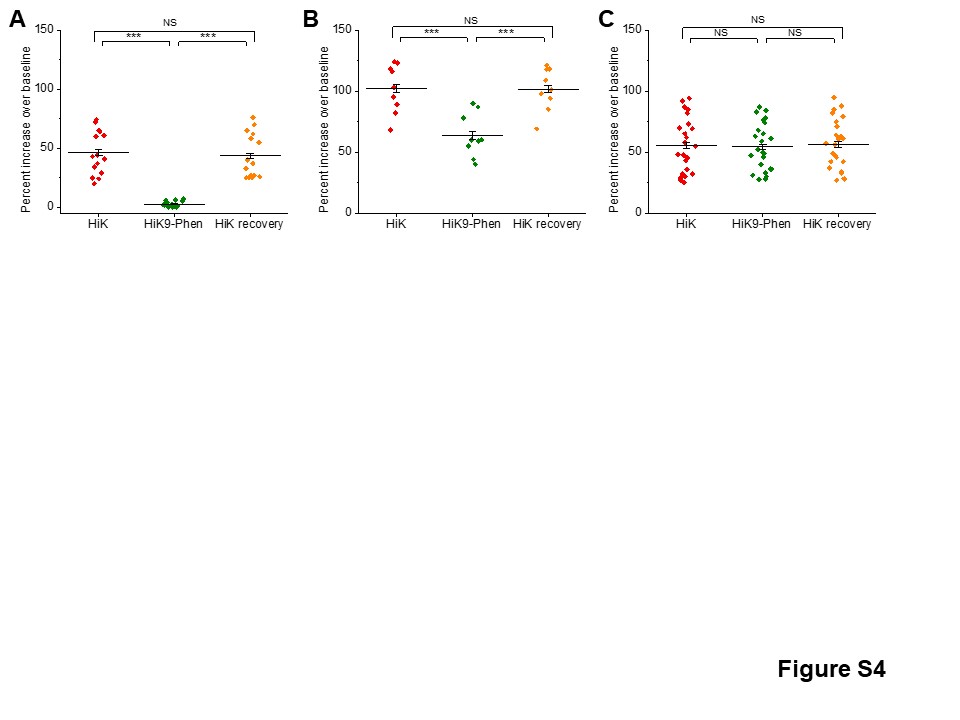

Supplement: Supplementary Figure 4 — Summary of the effects of 9-phenanthrol on VGCC activity in Type III cells. (A) Inhibition of TRPM4 abolished the calcium influx signals in some BR cells (F(2,42) = 38.5, p = 0.00000000031, n = 15 cells from 3 mice), while 9-phenanthrol partially inhibited calcium influx in other BR cells (B, F(2,24) = 12.9, p = 0.00015, n = 9 cells from 3 mice). (C) In other Type III cells, TRPM4 inhibition with 9-phenanthrol did not affect the calcium influx signals (F(2,69) = 0.54, p = 0.947, n = 24 cells from 7 mice) (***P < 0.001; N.S = Non-significant). [file Image_4.jpeg]

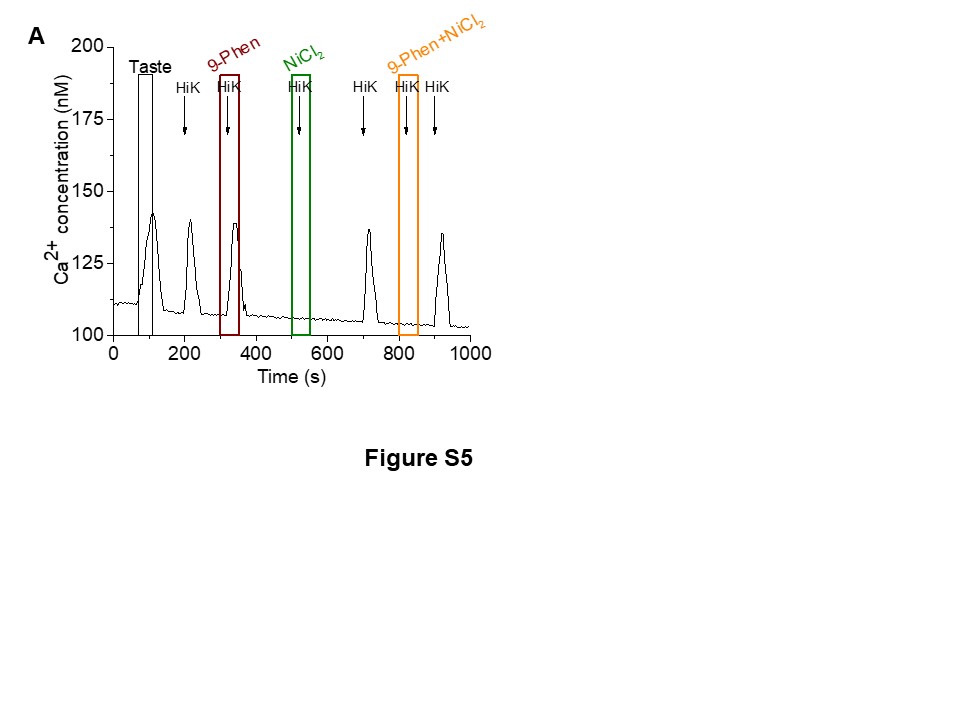

Supplement: Supplementary Figure 5 — TRPM4 does not interact with T-type VGCCs in BR cells. A representative calcium imaging trace showing that the calcium response generated by 30 mM KCl was not inhibited by 9-phenanthrol, however, this signal was completely inhibited by nickel chloride, a T-type VGCC blocker (n = 5 cells from 3 mice). [file Image_5.jpeg]

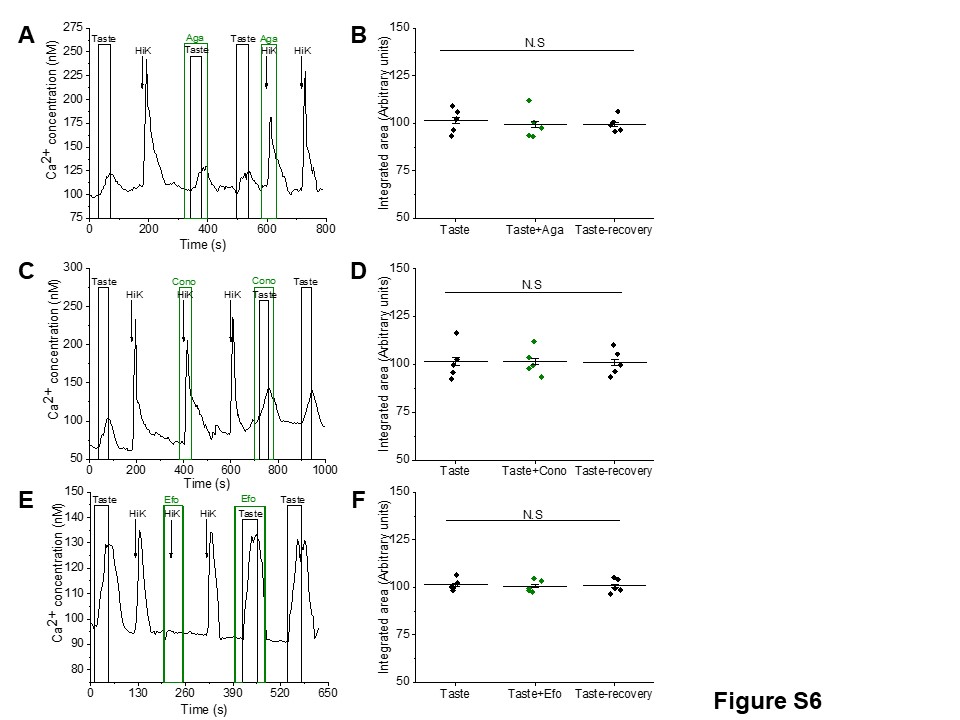

Supplement: Supplementary Figure 6 — Calcium influx through P/Q, N, and T-type VGCCs does not contribute to taste-evoke calcium responses in BR cells. (A,B) Application of ω-agatoxin IVA (300 nM) did not affect the magnitudes of the taste-evoked calcium responses in BR cells (F(2,12) = 0.17, p = 0.85, n = 5 cells from 3 mice). (C,D) Application of ω-conotoxin GVIA (800 nM) did not affect the magnitudes of the taste-evoked calcium responses in BR cells (F(2,12) = 0.004, p = 0.99, n = 5 cells from 3 mice). (E,F) Application of ephonididpine (20 μM) also did not affect the magnitudes of the taste-evoked calcium responses in BR cells (F(2,12) = 0.07, p = 0.94, n = 5 cells from 3 mice). [file Image_6.jpeg]

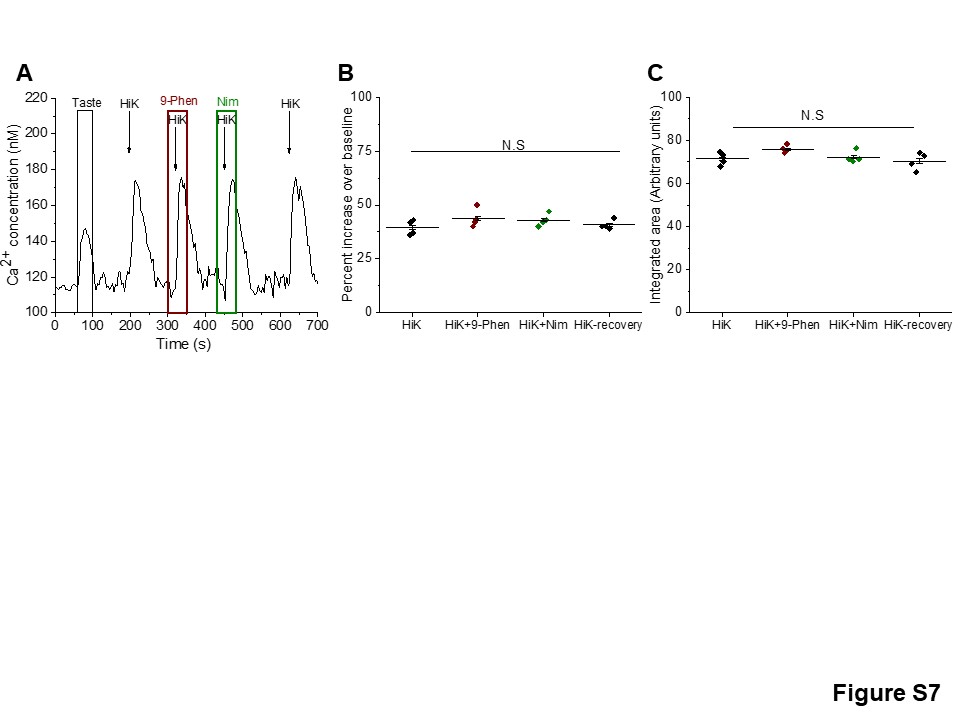

Supplement: Supplementary Figure 7 — BR cells are present in TRPM4-KO mice but 50mM KCl responses are no longer affected by inhibitors. (A) A representative calcium imaging trace of a BR taste cell from TRPM4-KO mice. In the TRPM4-KO BR cells, neither (B) the amplitudes nor (C) the magnitudes of the calcium signals generated by 50 mM KCl were inhibited by either 9-phenanthrol (50 μM) or nimodipine (10 μM) (amplitude; F(3,12) = 1.38, p = 0.30; magnitude; F(3,12) = 2.55, p = 0.10, n = 4 cells from 3 mice). [file Image_7.jpeg]
